# Application and Mechanism of Adipose Tissue-Derived Microvascular Fragments in Tissue Repair and Regeneration

**DOI:** 10.3390/biom15030422

**Published:** 2025-03-17

**Authors:** Yu Gao, Cheng Liang, Bingqian Yang, Li Liao, Xiaoxia Su

**Affiliations:** State Key Laboratory of Oral Diseases & National Clinical Research Center for Oral Diseases & Engineering Research Center of Oral Translational Medicine, Ministry of Education & National Engineering Laboratory for Oral Regenerative Medicine & Department of Pediatric, West China Hospital of Stomatology, Sichuan University, Chengdu 610041, China; gaoyu223@stu.scu.edu.cn (Y.G.); liangcheng@stu.scu.edu.cn (C.L.); yangbingqian1@stu.scu.edu.cn (B.Y.); lliao@scu.edu.cn (L.L.)

**Keywords:** vascularization, tissue regeneration, adipose tissue-derived microvascular fragments, tissue engineering

## Abstract

One of the long-standing challenges in the field of tissue repair and regeneration is the rapid establishment of local microvascular circulation and restoration of perfusion at the site of defects or injuries. Recently, adipose tissue-derived microvascular fragments (ad-MVFs) have attracted increasing attention from researchers. Adipose tissue is rich in blood vessels, and significant progress has been made in the extraction and preservation techniques for microvascular fragments within it. Ad-MVFs promote tissue and organ repair and regeneration through three main mechanisms. First, they accelerate rapid and efficient vascularization at the injury site, enabling early vessel perfusion. Second, the stem cell components within ad-MVFs provide a rich source of cells for tissue and organ regeneration. Third, they play a role in immune regulation, facilitating integration with host tissues after implantation. The application methods of ad-MVFs are diverse. They can be directly implanted or pre-cultivated, facilitating their combination with various scaffolds and broadening their application scope. These properties have led to the wide use of ad-MVFs in tissue engineering, with promising prospects. This review demonstrates that ad-MVFs can serve as a reliable and highly feasible unit for tissue regeneration.

## 1. Introduction

Blood vessels, lined with endothelial cells and surrounded by pericytes, are supported structurally by the extracellular matrix [1]. It is well established that vascularization plays a crucial role in transporting oxygen and nutrients and nourishing tissues throughout the body. Vascularization consists of several processes: angiogenesis, vasculogenesis, vascular anastomosis, vessel sprouting, and arteriogenesis. Angiogenesis refers to the growth of new capillaries from existing ones due to changes in the cellular environment or metabolism and can occur via endothelial cell budding or intussusception, where tissue columns divide into multiple vessels [2,3]. Vasculogenesis is the initial organization of endothelial cells leading to blood vessel formation, occurring in the absence of any existing vascular system, often when mesenchymal cells differentiate into hemangioblasts [4]. Vascular anastomosis involves the fusion of two vascular segments to create a continuous lumen [5], which is evident in tissue engineering, where microvascular networks constructed in vitro are infused with blood after implantation and connection to host blood vessels [6]. Vessel sprouting involves endothelial cells responding to vascular stimulation, with tip cells leading migration and forming new connections that eventually link with the parent vessel’s cavity, establishing new blood flow patterns [7]. Neovascularization is a broader concept, and angiogenesis is a form of neovascularization, including arteriography [8]. Arteriogenesis, the remodeling of arteries from existing arterioles to guiding arteries after arterial occlusion, plays an important role in compensating for ischemic disease [9,10]. Vasculogenesis, unlike angiogenesis, pertains to embryonic blood vessel formation from endothelial progenitor cells or angioblasts, beginning with cluster formation that includes peripheral angiogenic cells and hematopoietic stem cells (HSCs) [11,12]. Additionally, signaling molecules that influence tissue repair and regeneration are disseminated via these networks, which are vital for organ development, function, and the maintenance of homeostasis. Effective vascular regeneration is critical for creating large 3D tissues in regenerative medicine, facilitating cell survival post-transplantation, organ growth, and integration with the host system [13,14,15]. Moreover, research has shown that the organ-specific heterogeneity of differentiated endothelial cells meets the unique demands of organs for maintaining homeostasis [16].

## 2. Current Approaches to Achieving Vascularization

### 2.1. Cell-Derived Substances Promote Vascularization

Before delving into vascularization strategies, it is important to understand angiogenesis, a process involving the growth of capillary-like vascular sprouts regulated by a series of angiogenic factors. Endothelial cell proliferation is a hallmark of microvascular sprouting, while endothelial cell migration sustains the growth of these expanding sprouts [17]. This process is initiated by vascular endothelial growth factor (VEGF), which triggers endothelial cell assembly. VEGF was delivered to the lower limb skeletal muscle of patients with peripheral artery disease using a therapeutic-mediated cytokine delivery strategy, and VEGF was shown to modulate endothelial function and lower limb flow reserve in patients with peripheral artery disease [18]. Platelet-derived growth factor-BB (PDGF-BB) recruits pericytes and smooth muscle cells to further assemble the vasculature. Additional growth factors, such as angiopoietin-1 (Ang1) and transforming growth factor-beta 1 (TGF-β1), stabilize the newly formed blood vessels. Furthermore, proteolytic enzymes and their inhibitors, such as the plasminogen activator system and matrix metalloproteinases (MMPs), influence angiogenesis. Cell adhesion molecules play pivotal roles in this process as well [19,20]. Therefore, the use of cytokines is one of the important strategies to promote vascularization.

Similarly, significant progress has been made using biological agents or matrices to promote angiogenesis. Extracellular vesicles (EVs) derived from mesenchymal stem cells (MSCs) have been widely applied in tissue regeneration. EVs derived from umbilical cord mesenchymal stem cells can achieve rapid cell proliferation and migration by creating a satisfactory microenvironment and producing less immune response. Some studies have implanted the small intestinal submucosa (SIS) membrane modified by fusion peptide-mediated EVs with accurate spatial structure and excellent biocompatibility into rat abdominal wall defects, promoting tissue regeneration [21]. Recent studies have also shown that Forkhead box O1 (*FoxO1*) overexpressed small extracellular vesicles (sEVs) were used in periodontal tissue regeneration, demonstrating the ability to regulate osteogenesis and immune regulation [22]. Research has shown that EVs from human hypoxic olfactory mucosa MSCs (OM-MSCs) promote angiogenesis via *miR-612* [23]. Another study revealed that EVs loaded with VH298 can enhance vascularization by activating the HIF-1α signaling pathway [24]. Erythropoietin (EPO), primarily a regulator of erythropoiesis, also affects endothelial cells and nitric oxide synthase during wound healing, leading to increased expression of both. This continuous supply of nitric oxide (NO) concurrently elevates VEGF levels in wounds, thereby promoting angiogenesis [25].

### 2.2. Drug and Biomaterial Strategies That Promote Vascularization

Among pharmacological approaches, studies have found that vitamin D3 enhances angiogenesis by upregulating hypoxia-inducible factor 1-alpha (*HIF-1α*), which subsequently increases stromal cell-derived factor 1 (*SDF1*) expression. SDF1 receptor protein CXCR4 recruits angiogenic bone marrow cells, which play a role in vascular regeneration [26].

Biomaterials have also been shown to enhance vascularization by upregulating the expression of growth factors. For example, polyoctanediol–citrate–polyethylene glycol (POCG) copolymers facilitate endothelial vascularization by increasing the expression of angiogenesis factors through macrophage polarization-mediated mechanisms [27].

In addition to the individual effects of drugs, growth factors, cellular matrices, and materials, their combined application in promoting vascularization has been extensively explored. For instance, the combination of a conditioned medium (CM) derived from hypoxia-preconditioned mesenchymal stem cells and a soluble microneedle (MN) patch successfully promoted perifollicular vascularization [28]. The addition of *VEGF* plasmids to progenitor cell-derived exosomes promoted the vascularization process of bone tissue defect repair and regeneration [29]. Encapsulating VEGF within the core–shell structure of hierarchical micro/nanofiber biomimetic periosteum allowed its sustained release in fibrous layers and periosteum defect areas, significantly enhancing angiogenesis in the targeted region [30]. Furthermore, a novel dual-drug programmed release electrospun fiber mat (EFM) loaded with the angiogenesis promoter deferoxamine (DFO) and the osteogenesis inducer dexamethasone (DEX) demonstrated excellent vascularized bone formation capabilities [31]. In cases of myocardial infarction, VEGF-modified electrospun PGS–fibrinogen patches significantly improved vascular regeneration and the ejection fraction [32].

### 2.3. Cellular Strategies That Promote Vascularization

The essential role of cells in vascularization cannot be overlooked. Blood vessels are composed of multiple types of cells, making cell-based strategies a vital approach to supplying or producing these essential cells. Endothelial cells (ECs), the most crucial component of blood vessels, are typically cultured in three-dimensional gels composed of various extracellular matrix components to achieve vascularization [33,34,35,36,37,38,39,40]. Apart from transplantation using biological materials alone, ECs can be co-delivered with vascular growth factors via biomaterials to promote vascularization [41]. As previously mentioned, in addition to ECs, vascular components include pericytes. Interactions between ECs and pericytes jointly regulate the structure and function of microvessels. The co-culture of ECs and pericytes facilitates the assembly of vascular basement membrane matrices, stimulates matrix remodeling, and maintains stability. Co-culture with smooth muscle cells enhances the number of smooth muscle α-actin-expressing cells, which are crucial for human arteriole-like microvascular structures. After 60 days of co-culture, the number of large-diameter microvessels significantly increased [42,43]. Adult bone marrow tissues harbor various tissue-specific stem and progenitor cells, including endothelial progenitor cells (EPCs). Once EPCs enter circulation, they are termed circulating EPCs (CEPs). At ischemic sites, CEPs supplement pre-existing endothelial cells, contributing to neovascularization and facilitating rapid vascular reconstruction in damaged organs [44].

### 2.4. 3D Printing Technology Promotes Vascularization

Three-dimensional bioprinting is now widely used to create functional tissue structures with complex geometric shapes [45]. This technique is designed to replace or regenerate damaged tissues and organs [46]. There are many technologies for 3D bioprinting, among which laser-induced forward transfer (LIFT) stands out. It uses lasers to precisely transfer cells or bio-inks onto substrates, enabling the creation of complex 3D structures with high printing accuracy, enhanced cell survival, and excellent technical adaptability [47]. A study used this technique to prepare ECs into tubular structures with diameters of tens of capillary-like formations [48]. In short, innovations in 3D printing have further expanded vascularization research. Four categories of vascularized 3D-printed scaffolds have been identified: functional vascularized scaffolds, cell-based vascularized scaffolds, scaffolds loaded with specific carriers, and bionic vascularized scaffolds [49]. The lack of surface-active groups reduces the ability of scaffolds to vascularize. The introduction of bioactive groups is an effective way to imbue an inert surface with the desired biological properties. Surface functionalization methods reported so far include metals [50,51], active drugs [52,53], proteins or peptides [54,55,56], and growth factors [57,58,59]. This strategy is called functional vascularized scaffolds. The second strategy, cell-based vascularized scaffolds, refers to the inoculation of seed cells on scaffolds that allow the construction of bioactive tissue-engineering scaffolds capable of rapid vascularization [60]. It is mainly divided into three categories: 3D printed scaffolds with “cell” modifications [61,62], in which microvessels have excellent injectable and sutural properties and can be effectively introduced into 3D printed implants to promote tissue vascularization [63]. Also included are 3D printed scaffolds with composite cell inks [64] and 3D printed scaffolds with “gene-” modified cell inks [65]. Scaffolds loaded with specific carriers refer to the combination of active small molecules with 3D printed scaffolds through suitable carriers to obtain sustained and stable controlled release properties and endowing scaffolds with vascularization capabilities [66]. Scaffolds loaded with specific carriers can be roughly divided into three categories: hydrogels [67], microspheres [68], and nanoparticles (liposomes, exosomes, etc.) [29,69]. Finally, there are bionic vascularized scaffolds. First, “bionics” refers to an interdisciplinary field that combines biology and technology to study how the structure and function of living organisms work and invent new devices, tools, and techniques based on these principles to develop advanced technologies for production, learning, and living [70]. In response to the great challenge of the vascularization of tissue-engineering stents, bionic stents came into being, which can be divided into three categories: 3D printed scaffolds (“shells”) and hydrogels (“cores”) [71], 3D printed scaffolds (“shells”) with hollow tubes (“cores”) [72], and 3D printed scaffolds (“shells”) with blood vessel tips (“cores”) [73].

## 3. Adipose Tissue Contains Abundant Ad-MVFs, Which Have Strong Potential to Promote Tissue Repair and Regeneration

This review primarily investigates the effects and mechanisms of ad-MVFs in promoting tissue repair. Prevascularization strategies involve the use of microvascular fragments in three-dimensional induction cultures. Ad-MVFs, as natural vascularization units in angiogenesis and tissue-engineering research, represent powerful angiogenic agents. They are abundant and can be efficiently harvested from lipoaspirate or chunks of adipose tissues that have been cut off [74,75]. These fragments contain various types of cells [76], growth factors, and structurally intact vascular fragments, which can rapidly reassemble into microvascular networks [77]. These features lay the foundation for their broad application potential. This review discusses the extraction, cultivation, storage, and applications of ad-MVFs, exploring their effects and mechanisms in tissue repair and regeneration, as well as evaluating their feasibility for clinical applications.

### 3.1. Extraction of Ad-MVFs

Compared to stromal vascular fraction (SVF) isolation [78], ad-MVFs extraction requires less time, and ad-MVFs’ microvascular structure enables earlier in vivo implantation. The primary method for extracting ad-MVFs involves enzymatic digestion combined with mechanical separation. The microvascular fragments obtained mainly consist of small arteries, small veins, and capillaries [79,80,81]. The method used to extract ad-MVFs has been discussed for many years. The adipose tissue blocks are mechanically cut and digested with collagenase. The digested extracts are then subjected to gradient filtration, the effect of which is to remove single cells and small clusters of cells [39]. The extraction process has also been improved in recent years. Some teams have standardized the extraction scheme and elaborated the time of the enzyme digestion process. Longer digestion results in the tissue being digested into single cells without ad-MVFs, so the digestion time should be controlled within 10 min. The use of a 500 µm cell sieve can remove the remaining fat clots, while the use of a 20 µm filter can remove single cells in ad-MVFs, making it easier to focus on ad-MVFs experiments [80]. Comparing the extraction process of ad-MVFs and ADSCs, there are two distinct aspects of digestion time and the filtration procedure. The extraction of ADSCs requires a longer digestion time (45 min) compared to that of ad-MVFs (10 min). In addition, the filtration procedure of ADSCs and ad-MVFs is different with respect to the diameter of the final filter. ADSCs are isolated from cell suspension after passing through a 40 µm cell filter. Ad-MVFs are collected from the upper chamber of a 20 µm filter after the removal of single cells [76]. These fragments exhibit lumen-like structures and demonstrate a microvascular morphology that stabilizes surrounding cells [79,82]. Ad-MVFs extracted from *rat* [83,84] and *mouse* [85] adipose tissues are typically from visceral adipose tissues in the epididymal region. However, clinically accessible adipose tissue from *humans* is primarily subcutaneous adipose tissue.

The quality of extracted microvascular fragments is widely believed to depend on the quality of the source adipose tissue. Comparisons of microvascular fragments from obese and lean *mice* have shown that although the extraction density is lower in obese *mice*, more connective tissue is removed under the same separation conditions, and the resulting microvascular fragments are longer and exhibit better vascularization capacity [86].

The digestion time required to extract ad-MVFs from *human* adipose tissue is 1–2 h, which is longer than to extract ad-MVFs from *mouse* adipose tissue. At this time, elastic fibers in subcutaneous fat can be well degraded, so ad-MVFs are not lost during filtration, and ad-MVFs do not lose their complete vascular morphology [75,77]. However, research on the extraction of ad-MVFs from *human* adipose tissue remains limited. Factors such as age, sex, and anatomical location, as well as differences between brown and white adipose tissue-derived ad-MVFs require further investigation. These factors are crucial for advancing the clinical application of ad-MVFs.

### 3.2. Preservation of Ad-MVFs

For short-term storage, ad-MVFs preserved in a University of Wisconsin (UW) solution for 24 h do not exhibit significantly reduced vascularization capacity. Moreover, storing ad-MVFs at 4 °C in a UW solution enhances scaffold vascularization and tissue integration compared to storage at 20 °C [87]. For longer-term storage, freezing ad-MVFs for seven days has been shown to reduce transplantable ad-MVFs due to freezing and cell necrosis. However, the remaining ad-MVFs exhibit sufficient regenerative activity to compensate for the loss, supporting the feasibility of freezing as a storage method for ad-MVFs [88].

### 3.3. Differences in Blood Vessels Extracted from Adipose Tissue of Various Sites

Research on ad-MVFs extraction in *mice* has revealed that visceral adipose tissue yields a greater number of ad-MVFs compared to subcutaneous adipose tissue. Furthermore, implants seeded with visceral ad-MVF isolates exhibit significantly better vascularization compared to those seeded with subcutaneous ad-MVF isolates. This difference is attributed to the higher content of undigested connective tissue in subcutaneous ad-MVF isolates, which clogs scaffold pores and prevents individual ad-MVFs from connecting to form new microvascular networks [89]. However, subcutaneous fat is the primary adipose tissue available for clinical applications. Thus, improving methods to thoroughly remove connective tissue from extracts is critical for advancing the clinical application of ad-MVFs. Additionally, the extraction of ad-MVFs from different subcutaneous fat locations in *humans*—such as the thighs, abdominal region, and face/neck—remains an area with significant research potential regarding the quantity, quality, and activity of the extracted fragments.

One study compared the vascularization potential of ad-MVFs from male and female donor *mice*, specifically from epididymal and peri-ovarian adipose tissues. The results showed no significant differences in the number, length distribution, viability, or cellular composition of freshly isolated ad-MVFs between male and female donors. Additionally, ad-MVFs from both sexes exhibited similar in vitro sprouting activity after forming spheroids, as well as comparable vascularization and integration capabilities when implanted in vivo. These findings suggest that sex does not specifically influence the vascularization potential of ad-MVFs [90].

Systemic diseases, such as chronic diabetes, might affect the quality of extracted ad-MVFs. For example, diabetes can cause severe microvascular dysfunction, significantly impairing systemic microvascular function [91]. Additionally, factors such as age, body composition (obesity or leanness), and other individual differences merit further investigation to understand their impact on the quality of ad-MVFs.

## 4. The Role of Ad-MVFs in Tissue Regeneration

The mechanism by which adipose tissue-derived microvascular fragments promote tissue and organ repair is largely attributed to their components. Therefore, the discussion here focuses on the roles of various components. When discussing ad-MVFs, it is inevitable to mention SVFs, which are mixtures of various cell types extracted from adipose tissue. SVFs contain mesenchymal stem cells (MSCs), endothelial cells, fibroblasts, monocytes, and macrophages [92,93]. Compared to SVFs, which require longer enzymatic digestion times due to their single-cell suspension nature [94,95], ad-MVFs can be extracted with shorter enzymatic digestion. Component analysis of ad-MVFs shows the expression of endothelial cell markers (ILB4 and vWF), smooth muscle markers (α-SMA), and mesenchymal cell markers (CD44 and vimentin). Additionally, MSC markers, such as CD29, CD44, and CD90, are positively expressed in ad-MVFs, whereas another MSC marker, CD105, is not expressed. Endothelial progenitor cell (EPC) markers, including CD31 and CD34, are also expressed in ad-MVFs, with CD34 expression significantly higher than in SVFs. Moreover, hematopoietic cell marker CD45 is positively expressed in ad-MVFs [96].

**Figure 1:**
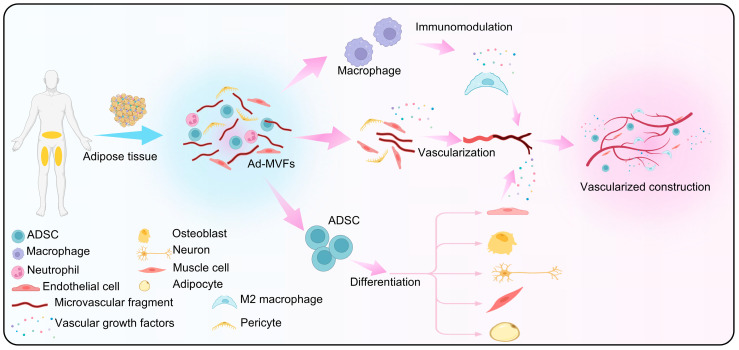
Ad-MVF components promote tissue repair and regeneration by promoting vascular regeneration, accelerating vessel perfusion, and providing abundant cell sources for rapid integration with target tissues.

In the process of promoting tissue repair and regeneration, ad-MVF components mainly play a major role. They promote the repair and regeneration of tissues by promoting vascular regeneration, accelerating tissue perfusion, and providing abundant cell sources for rapid integration with target tissues (Figure 1). At the same time, the synergistic effect with cells also further promotes the repair and regeneration of the tissue itself.

### 4.1. Ad-MVFs Promote Angiogenesis of Damaged Tissue

**Figure 2:**
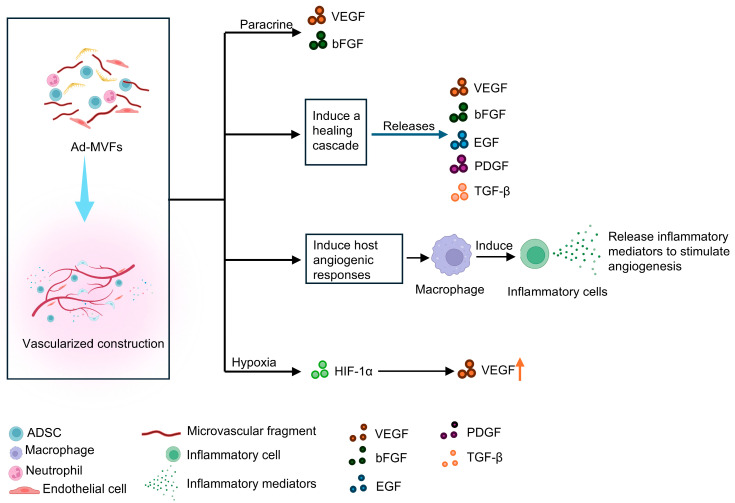
The main molecular mechanisms of promoting tissue vascularization after implantation of ad-MVFs: (1) implant paracrine action, (2) indirect activation of host cells, (3) activation of local immune cells, and (4) hypoxia-inducing factor signaling pathway.

**Figure 3:**
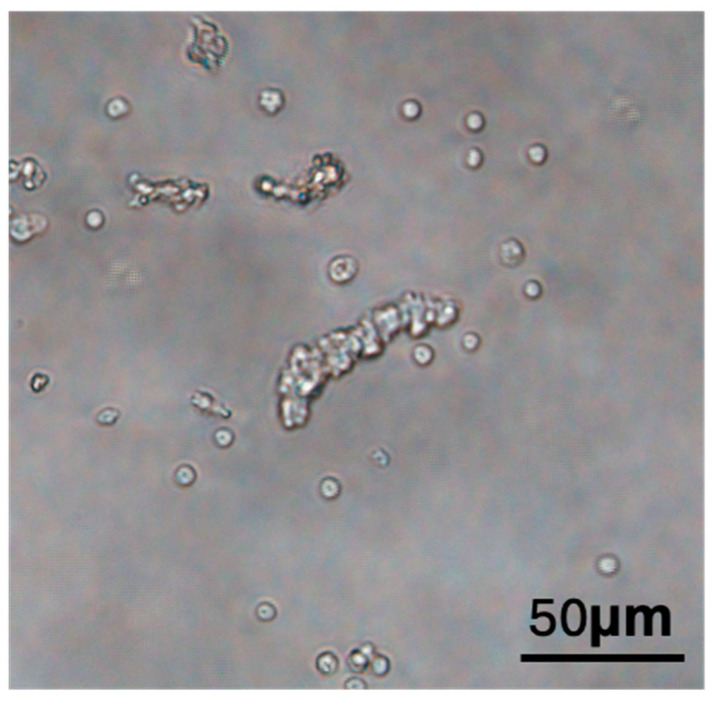
Appearance of extracted ad-MVFs under the light microscope.

We summarized some of the mechanisms that promote tissue vascularization after the implantation of ad-MVFs (Figure 2). First, microvascular fragments, as the protagonists of ad-MVFs, occupy a dominant position. Microvascular fragments play a central role in tissue repair due to their structural and functional characteristics. The length of microvascular fragments extracted and filtered typically ranges from 40 to 180 μm [97]. Microvascular fragments in ad-MVFs consist of segments of arteries, capillaries, and veins [85]. These vascular segments exhibit intact microvascular morphology (Figure 3), including lumens, endothelial cells, and perivascular cells [79,98]. Such features enable microvascular fragments to interconnect and integrate with surrounding host microvasculature after implantation, forming functional, perfused microvascular networks [82]. In this study, the researchers inoculated GFP-positive ad-MVFs onto a scaffold to implant the host and performed tissue immunofluorescence tests 14 days later. They found that 60% of CD31-positive microvessels in the host tissue surrounding the implant were GFP-positive, indicating that microvessels grew from the implant into the host tissue. In tissue engineering, prevascularization ensures rapid and adequate vascularization after implantation at the site of the host defect through an anastomosis process [6]. The preconceived condition of anastomosis between the two is to activate the host tissue microvessels to form capillary buds and grow to the preformed microvessels of the constructed graft. After transplantation, a classical healing cascade is induced, which releases large amounts of proangiogenic growth factors, VEGF, bFGF, EGF, platelet-derived growth factor (PDGF), and transforming growth factor-β (TGF-β) [99,100]. In addition, the implanted tissue construction itself may induce angiogenic host tissue responses [101]. The foreign body response triggered by the host after implantation generates a layer of host proteins on the surface of the material, and macrophages interact with this layer of proteins to release cytokines that activate other inflammatory cells [102]. These activated cells release inflammatory mediators, such as tumor necrosis factor, that directly stimulate the angiogenic process of tissue building [103,104,105]. Hypoxia during the initial stage of implantation in the host also leads to *HIF-1α* and the HIF-1α-mediated overexpression of *VEGF* [106]. In addition, anastomosis between the implant and the host tissue is divided into two forms, namely, “internal inosculation” and “external inosculation”, depending on where the vascular anastomosis occurs. Internal anastomosis occurs within a prevascularized graft due to the degeneration of the prevascularized microvessels and the invasion of host-derived microvessels along the previously designed graft vascular channels toward ischemic stimulation. The strong angiogenic activity of the graft prevascularization network may also cause the preformed blood vessels to grow outward from the graft into the surrounding host tissue, where external anastomosis occurs. At the same time, the preformed vascular endothelial cells contribute to angiogenic budding in the center of the graft and further increase the original microvascular density of the preformed microvascular network [6]. On this basis, by combining ad-MVFs, vascularization of the implants can be promoted. On the one hand, these ad-MVFs show good cell viability and a high ability to form functional microvascular networks within the implants. On the other hand, they also release VEGF and bFGF, which promote vascular growth, stimulating angiogenesis at the implant site [82].

Ad-MVFs also interact with surrounding cells. They contain endothelial cells and pericytes, which can synthesize type IV collagen and several laminin variants similar to those produced by aortic and lung endothelial cells, suggesting that pericytes are essential for the formation of the basement membrane, which is important for the regulation of endothelial cell migration and proliferation, as well as the tissue construction of microvascular networks [107]. In addition, ad-MVFs can secrete VEGF and bFGF [82]. It was previously known that the VEGF signaling pathway is a master regulator of angiogenesis. In short, endothelial cells express VEGFR-2, a tyrosine kinase receptor that positively drives endothelial cell proliferation and chemotactic responses to VEGF. Apical cell migration is dependent on the VEGF gradient, while stem cell proliferation is regulated by VEGF concentration. The leading apical cells respond to the VEGF gradient by migrating outward from the mother vessel and up the gradient. As blood vessels lengthen, stem cells proliferate in response to VEGF, form lumens, synthesize basement membranes, and associate with pericytes, increasing the mass and surface area of growing blood vessels [108,109]. bFGF is a potent mitogen of vascular and capillary endothelial cells in vitro and can stimulate the formation of blood capillaries in vivo (angiogenesis), which can excite the processes characteristic of angiogenesis in vivo, including endothelial cell migration, invasion, and plasminogen activator production [33].

Additionally, studies have identified lymphatic structures derived from implanted ad-MVFs, suggesting that ad-MVFs might also serve as building blocks for lymphatic networks within tissue-engineered scaffolds. These networks could reduce edema, clear local wound debris, and facilitate vascularization and integration with surrounding tissues [110].

Furthermore, microvascular fragments in ad-MVFs secrete vascular endothelial growth factor (VEGF) and basic fibroblast growth factor (b-FGF), indicating that they serve as a rich source of proangiogenic factors, stimulating angiogenesis in a paracrine manner [82,97]. The rapid formation of vascular networks, the paracrine secretion of cytokines that promote angiogenesis, and the ability to regenerate lymphatic vessels position ad-MVFs as highly efficient agents for early perfusion and vascularization at defect sites.

### 4.2. Ad-MVFs Promote Tissue Regeneration

A significant proportion of ad-MVF components consists of adipose-derived stem cells (ADSCs). These cells possess multipotency and active paracrine functions, which have demonstrated considerable potential in tissue repair and regeneration [111]. Therefore, we consider that ADSCs in ad-MVFs act in a similar way, when ad-MVFs are used to promote tissue regeneration. ADSCs share the morphological and immunophenotypic characteristics of mesenchymal stem cells (MSCs) [112]. When administered directly or combined with biofunctional scaffolds, ADSCs adhere, proliferate, and differentiate to replenish damaged tissues [113]. Originating from the mesoderm, ADSCs exhibit typical differentiation capabilities into adipogenic, osteogenic, and chondrogenic lineages. Remarkably, they can also differentiate into non-mesenchymal lineages, such as endothelial, myogenic, and neural lineages [113,114].

Techniques such as lineage-specific induction factors, mechanical or electromagnetic stimulation, and genetic reprogramming have been extensively used to induce ADSCs into specific cell types, highlighting their broad versatility and potential in regenerative medicine. Beyond their multipotency, the active paracrine functions of ADSCs further contribute to tissue repair and regeneration. ADSCs secrete VEGF, TGF-β, PDGF, angiopoietins (ANG), and other proangiogenic cytokines that collectively promote angiogenesis and facilitate tissue regeneration [115]. In addition, other studies have explored the ability of ADSCs to promote lymphopoiesis. Stimulation of ADSCs with VEGF-C in the short term leads to increased expression of *VEGF-A*, *VEGF-C*, and *Prox-1* in vitro, which is associated with significant lymphangiogenesis reaction after implantation in vivo. This lymphangiogenic response is significantly enhanced by blocking TGF-β1 function. In addition, the VEGF-C stimulation of ADSCs significantly increased cell proliferation and cell survival after implantation in vivo, and the stimulated cells expressed the lymphangiogenic cell marker podoplanin [116].

For damaged native tissues and newly formed tissues, ADSCs provide anti-apoptotic protection by secreting insulin-like growth factor-1 (IGF-1) and extracellular vesicles [117]. This advantage is particularly important in tissue-engineering applications, where ADSCs not only promote early vascularization but also extend the functional lifespan of implants and enhance their survival rates. Additionally, throughout the repair process, ADSCs exhibit immunomodulatory properties. They maintain local inflammatory responses necessary for repair while suppressing excessive immune reactions, regulating appropriate immune activity at the injury site, and thus promoting tissue regeneration [118,119].

Although we hypothesize that ADSCs in ad-MVFs also play the roles described above in tissue regeneration, more studies are needed to confirm their actual function.

### 4.3. Ad-MVFs Regulate the Immune Microenvironment for Tissue Regeneration

In addition, the extracted fat microvascular fragments contain macrophages, which secrete cytokines and growth factors facilitating inflammation and activating fibroblasts, tissue regeneration, and, finally, capsule formation [120]. They also stimulate collagen deposition after differentiation into multinucleated giant cells, which helps the implant to integrate with the host tissue quickly and effectively [121,122]. Moreover, it has been confirmed that ad-MVF/PRP-coated porous polyethylene (pPE), after implantation, induces an increase in CD163-positive M2 macrophages that promote angiogenesis in the surrounding host tissues and play a role in immune regulation [121], which is also the most important way for ad-MVFs to be involved in immune regulation. However, PRP has been shown to stimulate the polarization of macrophages, and the M2 macrophages detected 4 weeks after implantation are higher around PRP-coated and PRP/MVF-coated implants. It was not clear whether the decrease at 8 weeks was due to decreased PRP activity or because the local acute inflammatory response period had passed. In this study, several multinucleated giant cells were also found at the junction between the implant and the host tissue, of which 14 ± 3% were GFP+, indicating that their source was single monocytes or macrophages in the inoculated ad-MVFs. Therefore, the specific regulatory mechanism of ad-MVFs in immune regulation needs more relevant studies to be clarified. In addition, it has been confirmed macrophages themselves can not only regulate the immune function but also secrete various angiogenic growth factors, guiding endothelial tip cells, and participating in the interconnection of single angiogenic buds [123].

## 5. Strategies for the Application of Ad-MVFs

**Figure 4:**
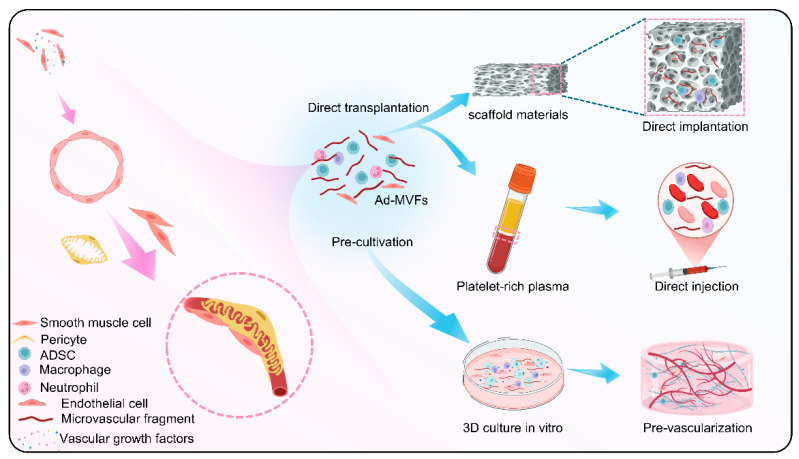
The process of angiogenesis and the strategies of ad-MVF application with direct transplantation and pre-cultivation.

The application of ad-MVFs can be divided into two categories. One is direct transplantation, including the use of scaffold materials for loading and injection in the form of liquid. The other type is pre-cultured in vitro and then implanted into the host tissue (Figure 4).

### 5.1. Application of Ad-MVFs to Facilitate the Repair and Regeneration of Tissues and Organs Without Cultivation

Polyurethane scaffolds seeded with microvascular fragments have been developed as a novel vascularization strategy focused on tissue engineering [124,125,126]. These polyurethane scaffolds, containing nano-sized hydroxyapatite particles, are characterized by their elasticity and high porosity, making them particularly suitable for the dynamic seeding of both single cells and microvascular fragments [82]. However, studies have indicated that pre-culturing in vitro with this setup does not significantly improve the in vivo vascularization capacity and might even alter the physiological morphology and function of the vessels. As a result, researchers concluded that using these scaffolds for immediate in vivo implantation is more suitable for promoting tissue repair and regeneration [127]. Like the polyurethane scaffold materials, some materials may not be suitable for pre-cultivation in vitro before implantation. However, they can serve as carriers for ad-MVFs and be directly applied to tissue defect repair and regeneration. For example, bioengineered dermal skin substitutes, designed for extensive skin tissue defect repair, can be used as scaffold materials. When ad-MVFs extracted from the adipose tissues of *mice* were seeded onto Integra, a collagen–glycosaminoglycan matrix, and implanted into a full-thickness dermal defect model in *mice*, significant results were observed. Compared to matrices without ad-MVFs, ad-MVFs quickly reassembled into microvascular networks within the implants, established connections with host microvessels, and formed lymphatic networks within the implants 14 days post-implantation. The degree of epithelialization in the defect area was also significantly better in the ad-MVFs group [79].

Further research by the same team applied this combination to a full-thickness dermal defect model in the cranial skin of *mice*. Ten days after the matrix was implanted, skin grafts were transplanted onto the matrix. The results showed that five days after transplantation, matrices seeded with ad-MVFs exhibited significantly higher graft survival rates compared to control groups [110]. When seeding ad-MVFs into scaffolds, the seeding density and the associated distance between fragments are critical for rapid vascularization. It was demonstrated that a density of 80,000 ad-MVFs/cm^2^ is the minimum requirement for effective vascularization of dermal substitutes [81]. Additionally, the scaffold itself must possess a porous structure to allow the continued expansion of the microvascular network formed by ad-MVFs.

A novel method for using ad-MVFs in the repair of tissue defects involves utilizing platelet-rich plasma (PRP) as a carrier. PRP can be quickly prepared from autologous blood samples through centrifugation or apheresis and contains a mixture of angiogenic growth factors. Compared to the use of recombinant growth factors alone, PRP promotes the formation of mature microvessels in a more physiological manner [128]. PRP transitions from an initial liquid form to a gel-like consistency after activation, making it adaptable to wounds of various shapes. It also serves as a natural carrier system for delivering additional combined cells [129,130]. Clinically, PRP is gaining increasing acceptance, with its safety and reliability being well-documented. Researchers have tested PRP as a carrier for ad-MVFs (isolated from *mice*) in the repair and regeneration of full-thickness dermal defects. Full-thickness skin wounds in the dorsal fold were made in *mice* and divided into three groups according to empty wounds (control), wounds filled with PRP alone, and wounds filled with PRP + MVF. Follow-up was 14 days after transplantation. The results indicated that wounds treated with PRP + ad-MVFs exhibited significantly accelerated and more effective healing. These wounds also demonstrated higher vascular and lymphatic vessel densities, effectively promoting granulation tissue formation at the site of the skin defect [131].

### 5.2. Three-Dimensional Culture of Ad-MVFs In Vitro Before Implantation

Angiogenesis begins with the assembly of endothelial cells, a process initiated by VEGF. Additionally, studies have shown that bFGF can stimulate characteristic angiogenic processes in vivo, including endothelial cell migration, invasion, and plasminogen activator production [33]. Therefore, VEGF and b-FGF are indispensable when inducing the vascularization of ad-MVFs in vitro.

To explore conditions that further enhance the vascularization potential of ad-MVFs in vitro, several studies have been conducted. For instance, it has been observed that stimulation of ad-MVFs with insulin-like growth factor 1 (IGF-1) for 24h improved their vascularization capacity before implantation [132]. Moreover, erythropoietin has also been shown to promote the vascularization of ad-MVFs in tissue-engineering applications [133]. Environmental conditions during in vitro cultivation also affect the vascularization capacity of ad-MVFs. For example, 24 h of subnormothermic cultivation at 20 °C preserved their viability better and enhanced proliferation and angiogenic activity compared to cultivation at 37 °C [134]. Additionally, in vitro culture in high-glucose solutions for 24 h significantly improved the vascularization potential of ad-MVFs in vivo.

Providing a physiological 3D extracellular matrix (ECM) environment is crucial for angiogenesis. Researchers have introduced a novel 3D culture system by embedding freshly isolated *rat* ad-MVFs in type I collagen gels [25]. This collagen gel culture environment is suitable for studying the effects of mechanical forces on microvascular network formation and orientation. By exposing the gel to a mechanical loading system, where it was anchored between a fixed pillar and a movable driving pillar, varying stress and strain fields were induced [135]. Under such mechanical regulation, it was found that ad-MVFs growth generally aligned parallel to the stress/strain direction or alonged internally generated traction forces, likely influenced by the alignment of surrounding collagen fibers [136]. Furthermore, the greater the constraining force applied to the boundaries of the collagen gel, the more strongly the angiogenic sprouting and branching of ad-MVFs were suppressed [137].

This suggested that appropriately applied mechanical forces could potentially guide vascular growth in a desired manner. Since vascular networks in different tissues exhibit diverse arrangements, this method could provide valuable insights for constructing vascular networks in organoids. Additionally, another study covered the surface of ad-MVFs/collagen gel with *rat* skeletal muscle satellite cells and found that this co-culture condition enhanced the angiogenic activity of ad-MVFs. However, when co-cultured with satellite cells from aged or dystrophic *mice*, the vascularization potential of ad-MVFs was diminished [138,139].

Furthermore, a team used a thermoresponsive hydrogel (TRH) as a carrier to deliver ad-MVFs for in vitro pre-cultivation and subsequent application to bone defect repair. This approach significantly promoted tissue vascularization. The TRH was found to maintain volume stability during gelation, preserve ad-MVF viability, and maintain vascular integrity. However, it may impair bone formation due to reduced *VEGF* expression during early bone healing and a non-physiological imbalance between RANKL and OPG during late bone healing [140].

## 6. Ad-MVFs Have Been Successfully Applied to Promote the Repair and Regeneration of Various Tissues and Organs

**Figure 5:**
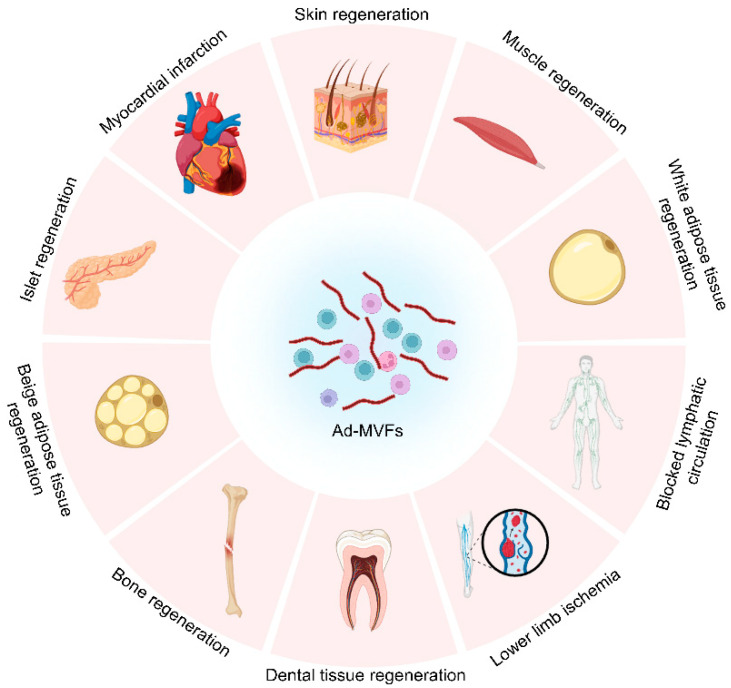
Ad-MVFs have been successfully applied to promote the repair and regeneration of various tissues.

Currently, ad-MVFs have been applied in the repair and regeneration of a variety of organs and tissues with different properties (Figure 5).

### 6.1. Applications in the Regeneration of Visceral Organs

In one study, microvessels (isolated from *mice*) were cultured in type I collagen in vitro for 7 days and then implanted into the infarcted myocardium of *mice*. Seven- and fourteen-days post-implantation, the transplanted microvessel grafts integrated well with the underlying cardiac tissue. Histological observations showed reduced fibrosis in the infarcted region, and the left ventricular function improved, demonstrating therapeutic effects for myocardial infarction [141]. Similarly, the prevascularization of isolated islets maintained the activity of islets after implantation in vivo and provided them with long-term survival [142]. In addition, compared with islets transplanted alone, the co-transplantation of islets and ad-MVFs significantly accelerated the normalization of blood glucose of diabetic recipients, indicating that the co-transplantation of ad-MVFs and islets could improve the success rate of islet transplantation [143].

### 6.2. Applications in Soft Tissue Regeneration

When ad-MVFs were seeded onto a collagen–glycosaminoglycan matrix and implanted into full-thickness skin defects in *mice*, the fragments demonstrated robust vascularization and lymphangiogenesis capabilities while accelerating integration with host tissues [79]. Furthermore, in applications for skin tissue regeneration, ad-MVFs combined with skin substitutes or transplanted skin flaps enhanced tissue repair and promoted integration between grafts and host skin [110,144,145]. Recently, a research team encapsulated *human*-derived ad-MVFs and fibroblasts in a composite hydrogel made of GelMA, HAMA, and fibrinogen using a 3D bioprinter. This approach successfully promoted epidermal regeneration, collagen maturation in dermal tissues, and skin vascularization, thereby accelerating wound healing [75]. For muscle tissue defects, ad-MVFs have also been shown to promote vascularization at the defect site [97]. However, for large-volume muscle defects, ad-MVFs did not exhibit significantly enhanced muscle tissue regeneration, indicating that other factors influence tissue regeneration despite local vascularization [146]. Additionally, ad-MVFs could serve as the sole biological material source for creating vascularized adipose tissue scaffolds, offering a promising method for soft tissue reconstruction [147].

### 6.3. Applications in Circulatory System Disorders

Intramuscular injection of microvascular fragments significantly increased blood flow and reduced tissue necrosis in ischemic limbs, demonstrating the potential of ad-MVFs for treating ischemic diseases [96]. Regarding lymphatic circulation, an essential component of the body’s circulatory system, the transplantation of ad-MVFs promoted angiogenesis and lymphangiogenesis, significantly increasing the density of microvessels and lymphatic vessels. These findings suggest that the transplantation of ad-MVFs is a promising therapeutic strategy for inducing lymphangiogenesis [148].

### 6.4. Applications in Metabolic Diseases

As one of the most prevalent clinical conditions, metabolic diseases have also been a focus of ad-MVFs-related research. Ad-MVFs isolated from adult patients can serve as a potential single autologous cell source to recreate beige adipose tissue functionality after induction. After 14 days of culture in vitro, these ad-MVFs achieved rapid vascularization, presenting a potential autologous therapy for metabolic diseases [149,150,151]. Recently, a research team utilized 3D printing technology to construct a vascularized islet encapsulation system combining β-like cells and ad-MVFs within a microdevice designed for the long-term therapeutic reconstruction of type 1 diabetes (T1D) [152].

### 6.5. Applications in Bone Regeneration

Blood vessels can transport cells, oxygen, nutrients, neurotransmitters, as well as growth factors, and hormones to bone tissue, thus playing an active role in bone formation and absorption [153]. Impaired blood vessel function and decreased perfusion can lead to the resorption of bone [154]. Therefore, the rapid establishment of vascularization is very beneficial and necessary in bone tissue repair and regeneration.

In bone tissue regeneration, thermoresponsive hydrogels (TRHs) loaded with ad-MVFs have demonstrated excellent regenerative effects [140]. Implanting ad-MVFs/GelMA hydrogels into *rat* cranial defects resulted in significant angiogenesis and osteogenesis [155]. In spheroids formed by osteoblasts and ad-MVFs, ad-MVFs reorganized into new microvascular networks, suggesting the potential of this strategy for bone tissue engineering [156]. In addition, the co-delivery of bone morphogenetic protein 2 (BMP-2) with ad-MVFs to bone injury sites associated with muscle injury has been shown to improve bone healing, manifested by a significant increase in regenerated bone volume and biomechanical properties [157]. In this study, the follow-up time after implantation was 12 weeks, which is the longest follow-up time for ad-MVFs in regenerative studies.

### 6.6. Applications in Dental Tissue Regeneration

We know that pulp plays a vital role in tooth growth and vitality, so a large part of the research on tooth regeneration focuses on how to promote pulp tissue regeneration. In dental regeneration, ad-MVFs have been found to effectively prevent apoptosis and senescence of transplanted dental pulp stem cells (DPSCs) both in vivo and in vitro. Ad-MVFs combined with DPSCs significantly promoted vascular network formation during transplantation, generated more odontoblast-like cells, and facilitated mineralization around the root canal [158]. In addition, ADSCs have been successfully demonstrated to grow tooth-like structures with nervous and vascular systems in adult *rabbits* and exhibit a stronger proliferation rate and superior ability to resist cellular senescence compared to dental pulp stem cells [159]. Compared with bone marrow mesenchymal stem cells (BMMSCs), which can be used in the regeneration of dental pulp, ADSCs transplantation produced more dental pulp-like tissues than BMMSCs transplantation, showing stronger regeneration potential [160]. As mentioned above, ADSCs in ad-MVFs mainly play a role in promoting regeneration, coupled with their role in promoting vascularization, so it is not difficult to see that ad-MVFs have strong potential to promote dental tissue regeneration.

## 7. Conclusions and Prospects

This article discusses the application of ad-MVFs, which can be broadly divided into three aspects: preparation before application, application mechanisms, and application effects. Preparation before application includes the extraction, storage, and in vitro cultivation of ad-MVFs. Although relatively mature extraction protocols exist, specific experimental evidence for achieving maximum efficiency in extraction is still lacking. Regarding storage, as previously mentioned, short-term storage can be accommodated; however, there is a lack of studies on long-term storage. In terms of in vitro cultivation, constructing an optimal 3D culture system for ad-MVFs is crucial. Particularly important thing is exploring conditions that promote the formation of vascular networks in vitro or even achieve perfusion. These factors can be categorized into the addition of exogenous biological factors to stimulate angiogenesis, the substrate conditions for 3D culture, and the environmental conditions.

Ad-MVFs contain a variety of cells that contribute to tissue regeneration and repair. Moreover, co-culturing ad-MVFs with different types of cells has provided insights into how cell–cell interactions enhance tissue regeneration. Notably, ad-MVFs offer the advantage of being derived from abundant sources without raising ethical controversies. They demonstrate effectiveness in promoting the repair and regeneration of various tissues and organs, highlighting their broad application potential. However, the specific mechanisms about how ad-MVFs play their roles after implantation remain an area for further study. For ad-MVFs’ cell components, such as stromal cells and ADSCs, it is difficult to find the relevant literature to track their migration and specific ways of effect after transplantation, which is also due to the excessive complexity of the components in ad-MVFs. Thus, more studies are needed to prove the role of specific components, which will help promote the further development of ad-MVFs.

It is known that endothelial cells, which form blood vessels, exhibit heterogeneity. Investigating this heterogeneity and its implications in the functionality of ad-MVFs will be essential for understanding their role in tissue regeneration and repair.

The heterogeneity of endothelial cells can be explained from two perspectives. First, differences exist among endothelial cells associated with different vascular types. As the innermost cellular lining of blood and lymphatic vessels, endothelial cells exhibit significant structural and functional variations [161]. Second, endothelial cells exhibit specific phenotypes in response to changes in their microenvironment [162]. Many studies have confirmed that endothelial cells originating from different tissues and organs possess organ-specific characteristics. A team has developed models for endothelial cell purification, culture, analysis, differentiation, and transplantation, establishing a database of microvascular heterogeneity [163]. Organ-specific endothelial cells also play unique roles in tissue and organ regeneration. For instance, sinusoidal endothelial cells in bone marrow support hematopoiesis by expressing Notchligands, EGF, pleiotrophin, and stem cell factor (SCF, Kit ligand) [164,165,166,167]. In the liver, sinusoidal endothelial cells promote liver regeneration after a 70% partial hepatectomy by expressing Wnt2 and hepatocyte growth factor (HGF) [168]. In lung tissues, endothelial cells support alveolar regeneration by providing MMP14 and EGF-like ligands [169]. These findings suggest that endothelial cells in each tissue and organ are unique and can promote regeneration in a tissue- and organ-specific manner.

Based on these studies, further research could explore whether endothelial cells in ad-MVFs also exhibit similar organ-specific properties. Additionally, it would be valuable to investigate whether ad-MVFs transform target organ-specific endothelial cells when used to promote the repair and regeneration of other tissues and organs. Understanding these mechanisms could provide insights into how ad-MVFs promote tissue regeneration and identify methods or conditions to enhance their regenerative capabilities. In addition, how other cellular components of ad-MVFs change after being cultured in vitro to implanted in vivo and how they relate to surrounding tissues still needs more research.

In the field of tissue regeneration, overcoming challenges related to vascular regeneration and the early establishment of local circulation remains a significant hurdle. Various vascularization strategies have been proposed. Among them, ad-MVFs are gaining recognition as a readily available, safe, reliable, and powerful strategy. Their sourcing involves minimal ethical concerns and represents a sustainable reuse of discarded surgical tissue materials. This is a significant advantage over all the other vascularization strategies mentioned above; in terms of acquisition sources, it realizes the conversion of waste into treasure and reduces the acquisition cost. The well-established extraction protocols and reliable storage methods highlight their potential for clinical translation. Additionally, the rich cellular composition of ad-MVFs endows them with strong vascularization capabilities and the ability to promote tissue and organ repair and regeneration. Compared with other vascularization strategies, which own relatively single cellular components, ad-MVFs contain a variety of cells, which can play a certain role in promoting vascularization, tissue regeneration, and immune regulation, implying that they have a comprehensive role in promoting tissue repair and regeneration. Numerous in vitro and in vivo studies have demonstrated their regenerative potential across various types of tissues and organs. They also have some disadvantages. For example, compared with 3D bioprinted vascularization strategies, the scale or volume of the vascular network formed by ad-MVFs in vitro may not have an accurate setting, so this point should be further studied in later studies. In addition, their rich cellular components may also mean that more experiments are needed to help develop the detection and specification of the extraction quality, such as the influence of sampling age, physical health status, etc., on different groups in ad-MVFs, what conditions can obtain the best quality ad-MVFs, etc. These need to be further clarified in the future.

However, several research areas remain to be explored. Specifically, mechanistic studies are needed to understand the changes that occur when ad-MVFs are applied to other tissues and organs. Questions such as whether endothelial cells within ad-MVFs undergo organ-specific phenotypic changes and how ad-MVFs interact with different tissues and organs require precise answers. Addressing these questions is critical for expanding the application prospects of ad-MVFs. Furthermore, transitioning to clinical trials will require in-depth investigations into extraction strategies, storage methods, and inclusion criteria. In addition, in future clinical application conditions, ad-MVFs will be transplanted into the tissue defects of patients, mostly through autologous transplantation, so it is necessary to have a clear understanding of the factors affecting the quality of ad-MVFs, which needs more research for clarification. In addition, the in vitro generation of artificial tissue substitutes with highly tissue-preformed blood vessels by ad-MVFs requires complex bioreactor systems that should allow microfluidics to incorporate microvascular fragments into the microstructural network topology, as well as achieve dynamic in vitro perfusion and maturation of the networks through these fragments [85].

In conclusion, ad-MVFs represent an excellent vascularization and regeneration strategy with significant potential for application in the repair and regeneration of tissues and organs.

## Data Availability

Not applicable.
